# Phase 3 Randomized Low-Dose Paclitaxel Chemoradiotherapy Study for Locally Advanced Non-Small Cell Lung Cancer

**DOI:** 10.3389/fonc.2016.00260

**Published:** 2016-12-20

**Authors:** Hongmei Lin, Yuhchyau Chen, Anhui Shi, Kishan J. Pandya, Rong Yu, Yannan Yuan, Jiancheng Li, Hang Li, Yingjie Wang, Tingyi Xia, Linchun Feng, Huimin Ma, Jianhao Geng, Guangying Zhu

**Affiliations:** ^1^Key Laboratory of Carcinogenesis and Translational Research (Ministry of Education), Department of Radiation Oncology, Peking University Cancer Hospital & Institute, Beijing, China; ^2^Department of Radiation Oncology, China-Japan Friendship Hospital, Beijing, China; ^3^Department of Radiation Oncology, James P. Wilmot Cancer Institute, University of Rochester Medical Center, Rochester, NY, USA; ^4^Division of Hematology and Oncology, Department of Medicine, James P. Wilmot Cancer Institute, University of Rochester Medical Center, Rochester, NY, USA; ^5^Department of Radiation Oncology, Fujian Province Cancer Hospital, Fuzhou, China; ^6^Department of Radiation Oncology, Guizhou Province People’s Hospital, Guiyang, China; ^7^Department of Radiation Oncology, Air Force General Hospital, PLA, Beijing, China; ^8^Department of Radiation Oncology, Chinese PLA General Hospital, Beijing, China

**Keywords:** non-small cell lung cancer, paclitaxel, chemoradiotherapy, radiosensitization, phase 3 trial

## Abstract

**Introduction:**

Concurrent chemoradiotherapy (CCRT) is the standard treatment for locally advanced non-small cell lung cancer (LA-NSCLC) but is associated with poor chest tumor control. Here, we report results of a randomized phase 3 study comparing two CCRT regimens in improving chest tumor control by low-dose paclitaxel chemoradiation for LA-NSCLC.

**Methods:**

Due to the logistics of local referral pattern, the study was designed to enroll patients with stage III LA-NSCLC who had completed 2–4 cycles of full-dose chemotherapy. One hundred thirty four were randomized to either Arm 1 [paclitaxel at 15 mg/m^2^, three times per week (Monday, Wednesday, and Friday) for 6 weeks, *n* = 74] or Arm 2 (weekly paclitaxel at 45 mg/m^2^ for 6 weeks, *n* = 60). Chest radiotherapy was 60–70 Gy in standard fractionation. Response rate was the primary endpoint, with recurrence-free survival (RFS) as the secondary endpoint.

**Results:**

From March 2006 to February 2013, 71 patients completed Arm 1 treatment and 59 completed Arm 2 treatment. The response rate for Arm 1 was significantly higher (83.1%) than Arm 2 (54.2%) (*p*=0.001). RFS was superior in Arm 1: median 14.6 vs. 9.4 months, *p* = 0.005, Hazard ratio (HR) 1.87 [95% confidence interval (CI) 1.20, 2.90]. Overall survival was not significantly different: median 32.6 months in Arm 1 vs. 31.3 months in Arm 2, *p* = 0.91, HR 0.97 (95% CI 0.55, 1.70). Toxicity was significantly lower in Arm 1 for Grade 3 and 4 leukopenia/neutropenia (*p* < 0.001).

**Conclusion:**

Pulsed low-dose paclitaxel CCRT resulted in significantly better RFS and tumor response rate, and less hematologic toxicities than weekly CCRT for LA-NSCLC.

## Introduction

Lung cancer is the leading cause of cancer-related deaths for both men and women worldwide (1) and is a serious and growing health problem in China, with approximately 600,000 deaths each year. The cancer rate has been rising in China since the early 1980s because of the increasing smoking population (2). Thoracic radiotherapy (RT) remains the standard and widely utilized treatment modality for inoperable, locally advanced non-small cell lung cancer (LA-NSCLC).

Randomized studies have demonstrated that concurrent chemoradiotherapy (CCRT) yielded better survival rates than the sequential approach but with higher treatment toxicities (3, 4). Besides efficacy, treatment toxicity does influence the treatment of choice by oncologists and patients (3, 4). Comprehensive literature reviews have shown that toxicity profile is more favorable in low-dose CCRT, but low-dose CCRT has not been widely adapted worldwide (5). In addition, in the era of CCRT, the chest tumor control rate has been disappointing, averaging less than 50% by radiographic criteria. Uncontrolled chest tumors (including both the primary and hilar/mediastinal lymph nodes) represent a major barrier to further therapeutic gain, as surviving cancer cells in residual chest tumors can seed distant metastases. In this context, the significance and the impact of chest local–regional tumor control for LA-NSCLC on survival outcome has been analyzed in 1,390 patients treated in 7 legacy Radiation Therapy Oncology Group (RTOG) trials by CCRT for LA-NSCLC (6). This analysis revealed that the chest local–regional tumor control rate at 3 years was 38% at best and only 14% using a more strict definition (7). This analysis did find a powerful association between chest local–regional tumor control and overall survival (OS), which was statistically significant (*p* = 0.0001). Such finding highlights the importance of chest tumor control on the survival outcome of LA-NSCLC.

Optimal CCRT in maximizing chest tumor control and minimizing toxicities remains to be defined. Taxane-based CCRT has been favored for the treatment of LA-NSCLC by many oncologists worldwide. Taxanes are known to have antiangiogenic effects on tumor vasculature (8, 9). These are also cell cycle-specific chemotherapeutic agents that can cause cytokinetic stabilization of the spindle microtubules leading to apoptotic cell death (10, 11). The cell cycle effect of paclitaxel on the arrest in the G2/M phase, the most radiosensitive phase, makes it an ideal radiation sensitizer to enhance RT effects (12). In the U.S., weekly paclitaxel-based CCRT has been published in many phase 1, 2, and 3 clinical trials for CCRT, and has been shown to be better tolerated than cisplatin-based CCRT (13, 14).

Weekly low-dose carboplatin and paclitaxel with concurrent radiation combination is a widely accepted standard in the U.S. and Europe for LA-NSCLC. Despite common practice, the optimal dose-schedule of paclitaxel for CCRT treatment of LA-NSCLC remains to be defined. Previously, a phase 1 and 2 clinical study was conducted using pulsed, low-dose paclitaxel CCRT for LA-NSCLC (15, 16). Based on preclinical investigations, it was hypothesized that a regimen of schedule-dependent, pulsed, low-dose paclitaxel CCRT could yield effective radiosensitization to improve chest tumor control. This clinical trial has reported promising tumor response rates and a high rate of infield tumor control with low toxicities (15–17). We also conducted a phase 1 clinical study testing this hypothesis-based study design in China (18). Following up the phase 1 study, we conducted a multicenter randomized phase 3 study comparing two low-dose sensitizing paclitaxel schedules of CCRT for LA-NSCLC. We hypothesized that a schedule-dependent, pulsed low-dose paclitaxel (15 mg/m^2^, three times per week) CCRT regimen would yield better recurrence-free survival (RFS) than the commonly applied weekly paclitaxel (45 mg/m^2^) radiation regimen due to more effective radiosensitization.

## Methods

### Patient Eligibility

This multi-institutional clinical study is registered with the Chinese Clinical Trial Registry (ChiCTR-TRC-10000786) and was conducted under the approved clinical protocol of the institutional review board at Beijing Cancer Hospital (RCOG-0701) and each participating hospital. At the time of this study, separate institutional review board reviews were not necessary for investigators from other hospitals to participate in the study. All patients gave written informed consent to study participation. Patients with histologically or cytologically confirmed inoperable stage IIIA or IIIB NSCLC were eligible. In our region in China, most patients with LA-NSCLC would have completed 2–4 cycles of chemotherapy before referral for chest RT. In consideration of the logistics of patient enrollment, patients who had completed 2–4 cycles of induction chemotherapy within 4–8 weeks prior to the referral for chest RT were eligible.

Patients needed to have at least one bidimensionally or unidimensionally measurable tumor <8 cm in size on CT imaging. Additional eligibility criteria included patient age of 18–75 years, <10% weight loss within 6 months, ECOG performance status 0–1, leukocyte count ≥4,000/μL, neutrophil count ≥1,500/μL, platelet ≥100,000/μL, hemoglobin ≥10 g/dL, creatinine ≤1.5 upper normal limit, total bilirubin ≤1.5 upper normal limit, AST and ALT ≤2.5 upper normal limit, alkaline phosphatase ≤5 upper normal limit, and FEV1 >50% predicted. Exclusion criteria included pregnancy, significant cardiac disease, uncontrolled diabetes, second primary tumor other than non-melanoma skin cancer or *in situ* cervical carcinoma, serious infections, uncontrollable psychoses, hypersensitivity to paclitaxel, or participation in another clinical trial. Clinical evaluation included weight, performance status, blood tests, pulmonary function test, and ECG. Radiographic staging included chest X-ray, ultrasound of upper abdomen and supraclavicular lymph nodes, computed tomography of chest, magnetic resonance imaging or computed tomography of the brain, and bone scan. Some patients had FDG-PET scans, but this was not a required test per the study protocol.

### Treatment Schema

The allowed induction chemotherapy included cisplatin-containing dublets: vinorelbine/cisplatin, gemcitabine/cisplatin, paclitaxel/cisplatin, docetaxel/cisplatin, or pemetrexed/cisplatin. Enrolled patients were randomly assigned to one of the two treatment arms using the published maximum tolerated dose (18). CCRT was initiated 4–8 weeks after completion of induction chemotherapy. Arm 1 received CCRT with three-times weekly paclitaxel (15 mg/m^2^, three times per week on Monday, Wednesday, and Friday, with the cumulative total of 270 mg/m^2^ in 6 weeks) and concurrent RT. Arm 2 received CCRT with the weekly paclitaxel (45 mg/m^2^, once/week, to the cumulative total 270 mg/m^2^ in 6 weeks). 3D-conformal chest RT or intensity-modulated RT were allowed. For Arm 1, daily RT was delivered at least 4 hours after paclitaxel infusion on Monday, Wednesday, and Friday to allow for cell cycle progression to the G2/M phase (15). For Arm 2, there was no stipulation of the timing of daily RT, which is common practice. All patients received standard premedication (dexamethasone, diphenhydramine hydrochloride, and cimetidine) before paclitaxel infusion to reduce the risk of hypersensitivity.

### Treatment Dose Modifications

Chemotherapy was discontinued if the granulocyte count was <0.5 × 10^3^/mL and/or the platelet count was <25 × 10^3^/mL and severe hypersensitivity occurred. Chemotherapy was delayed when the granulocyte count was <1.5 × 10^3^/mL and/or the platelet count <75 × 10^3^/mL on the day of chemotherapy. If the granulocyte counts remained 0.5–1.5 × 10^3^/mL or the platelet counts remained 25–75 × 10^3^/mL for more than 1 week, chemotherapy was discontinued.

### Radiotherapy

Radiotherapy treatment planning utilized CT simulations. Gross target volume (GTV) included the primary lesion and involved lymph nodes (1 cm or larger). Clinical target volume (CTV) was defined as the GTV plus a 0.6 or 0.8 cm margin, and the planning target volume (PTV) was defined as the CTV plus a 1–1.5 cm margin for setup uncertainty and respiratory motion. Regional lymph nodes were not electively irradiated. All patients received the prescribed dose (60–70 Gy/30–35 fractions/6–7 weeks) to 95% of the PTV. Dose limitation for organ-at-risk was defined: lung V20 < 30%, esophagus V55 < 50%, heart V40 < 40%, and <40 Gy for maximum spinal cord dose.

### Assessment of Efficacy and Safety

The primary endpoint of the study was response rate assessed by RECIST criteria (19). The secondary endpoints were RFS and treatment-related toxicities of the two treatment arms. Toxicities were evaluated weekly during CCRT and follow-up visits. Follow-up evaluation included history and physical examinations, CT scans of the chest, and blood tests 1 month post-CCRT and every 3 months thereafter. Local–regional responses to therapy were determined from the chest CT 1 month after the end of CCRT. Toxicity was monitored per Common Terminology Criteria for Adverse Events version 3.0 (20).

### Sample Size Estimate

Based on a two-sided Fisher’s exact test with a targeted significance level of 5%, sample sizes of 60 in each treatment group would achieve 76% power to detect a significant difference of 25% in response rates between the two arms if the response rates in Arm 1 and in Arm 2 were 75 and 50%, respectively. For the primary objective of comparing response rates between treatment arms, we planned to screen 134 (67 in each arm) and allow a 10% drop out rate to result in sufficient cases of 60 in each arm for the comparison of the primary endpoint.

### Randomization

In this study, we used the method of random number table, which belongs to the simple randomization and grouping the patients. We selected any number from the random number table and got next number in the same direction order for each patient. Each random number was divided by the number of groups (two in this study), then we grouped the patients according to the remainder. If the remainder was 1, the patient was enrolled into Group A; if the remainder was 0, the patient was enrolled into Group B. This method was much more effective, but the disadvantage was that the imbalance of results might exist. Guangying Zhu and statistician Yannan Yuan decided the method of randomization, randomly chose the first number from the random number table, and decided the right as the direction order. Anhui Shi and Hongmei Lin carried out the randomization and grouping, and participating physicians in the study were responsible for the enrollment and intervention of participants. This study was not blinded, as the treatment schema of each arm was self-evident of the randomized arms.

### Statistical Analysis

Clinical data were collected by the investigators of the participating hospitals and centrally collected by Hongmei Lin and Yannan Yuan. RFS and OS were defined from the start time of any cancer treatment (first cycle of induction chemotherapy) to last follow-up after CCRT, or death due to any cause for OS, and any recurrence/progression (local, regional, and distant) for RFS. The distribution of OS and RFS were estimated using Kaplan and Meier’s method. They were compared between arms using a two-sided log-rank test. Hazard ratios (HRs) and their 95% confidence intervals (CI) were constructed using Cox’s proportional hazard model. The comparison of response rates, patterns of failure, and the incidences of toxicities were analyzed using Fisher’s exact test and analysis of variance. Results were considered statistically significant at the 5% level (*p* < 0.05).

## Results

### Patient Characteristics

**Figure 1:**
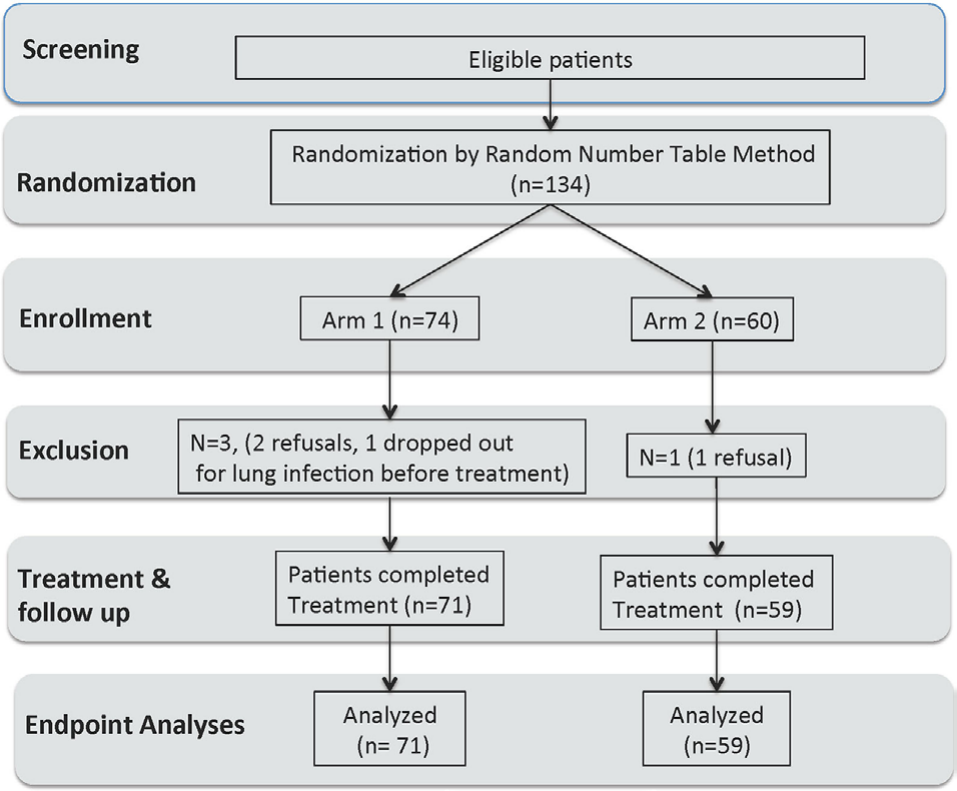
**Consort diagram of patient randomization and treatments**.

From March 2006 to February 2013, 134 patients were enrolled as planned. Random number table randomization resulted in 74 patients in Group A for Arm 1 and 60 patients in Group B for Arm 2. Among Arm 1 patients, three patients went off the study (two for subsequent refusal and one for pulmonary infection before protocol treatment). Among Arm 2 patients, one patient went off the study because of refusal. The final number of cases for statistical analysis was 130, with 71 in Arm 1 and 59 in Arm 2 (Figure 1). Despite some imbalance in each arm from randomization, the baseline patient and disease-related characteristics of the two arms were not significantly different (Table 1). This study did not do blinding as a result of the study design.

**Table 1:** **Comparison of baseline characteristics of patients who completed protocol treatments**.

	No. of patients		*p*-Value
	Arm 1 (*n* = 71)	Arm 2 (*n* = 59)	

Characteristics			
**Age (years)**			
Median	59	56	0.540
Range	22–75	36–75	
**Sex**			
Male	57 (80%)	50 (85%)	0.507
Female	14 (20%)	9 (15%)	
**Ethnicity**			
Han	70 (99%)	58 (98%)	0.745
Other	1 (1%)	1 (2%)	
**Performance status**			
0	43 (61%)	42 (71%)	0.205
1	28 (39%)	17 (29%)	
**Histology**			
Squamous	42 (59%)	41 (69%)	0.123
Adenocarcinoma	23 (32%)	17 (29%)	
Large-cell	4 (6%)	0	
Adenosquamous	2 (3%)	1 (2%)	
**Stage**			
IIIA	25 (35%)	16 (27%)	0.323
IIIB	46 (65%)	43 (73%)	
**Weight loss**			
≤5%	63 (89%)	48 (81%)	0.236
>5%	8 (11%)	11 (19%)	

### RFS and OS

**Figure 2:**
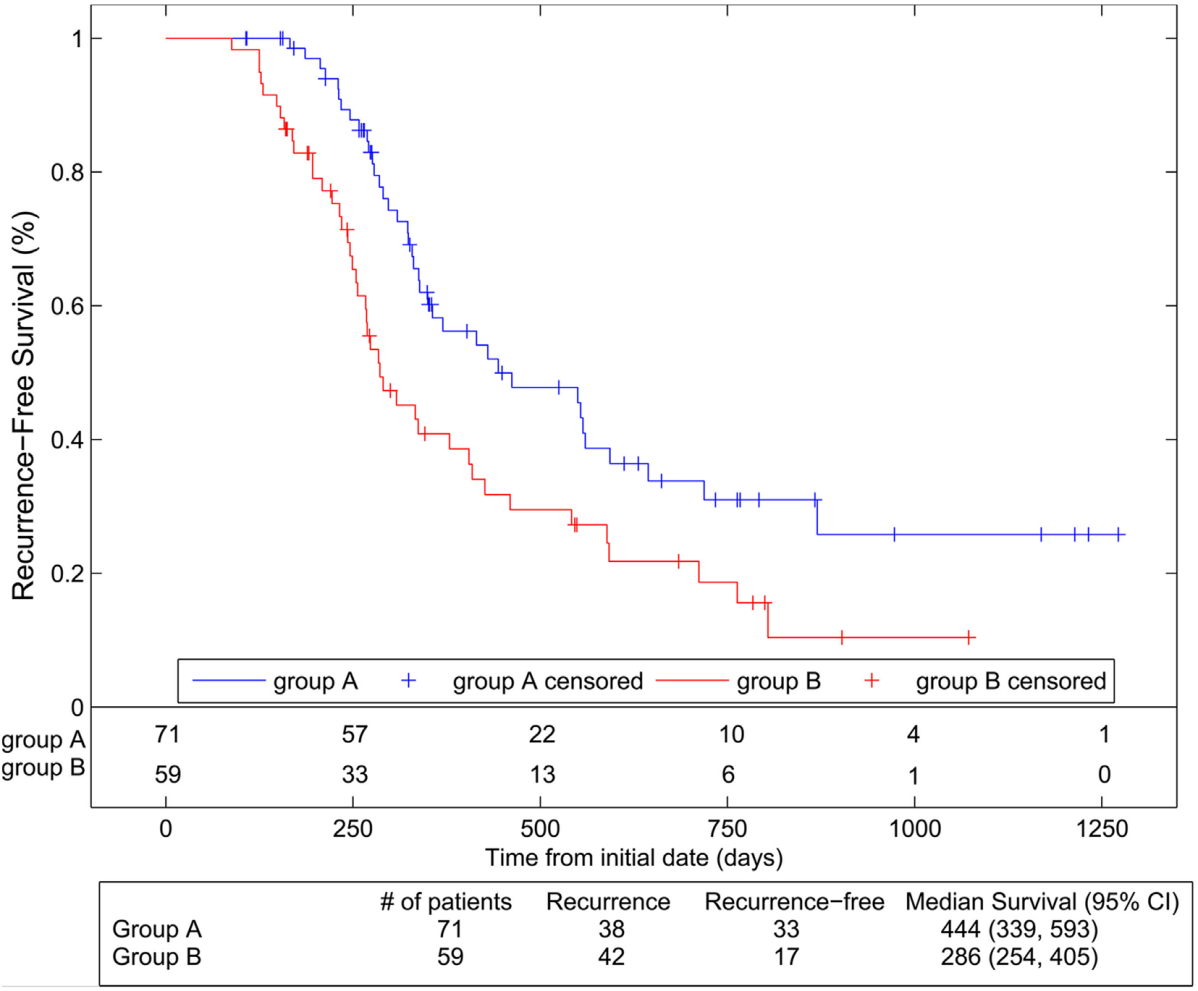
**Recurrence-free survival compared between the two arms using Kaplan–Meier analyses**. Group A represents Arm 1 (paclitaxel 15 mg/m^2^, three times per week), and Group B represents Arm 2 (paclitaxel 45 mg/m^2^ per week).

**Figure 3:**
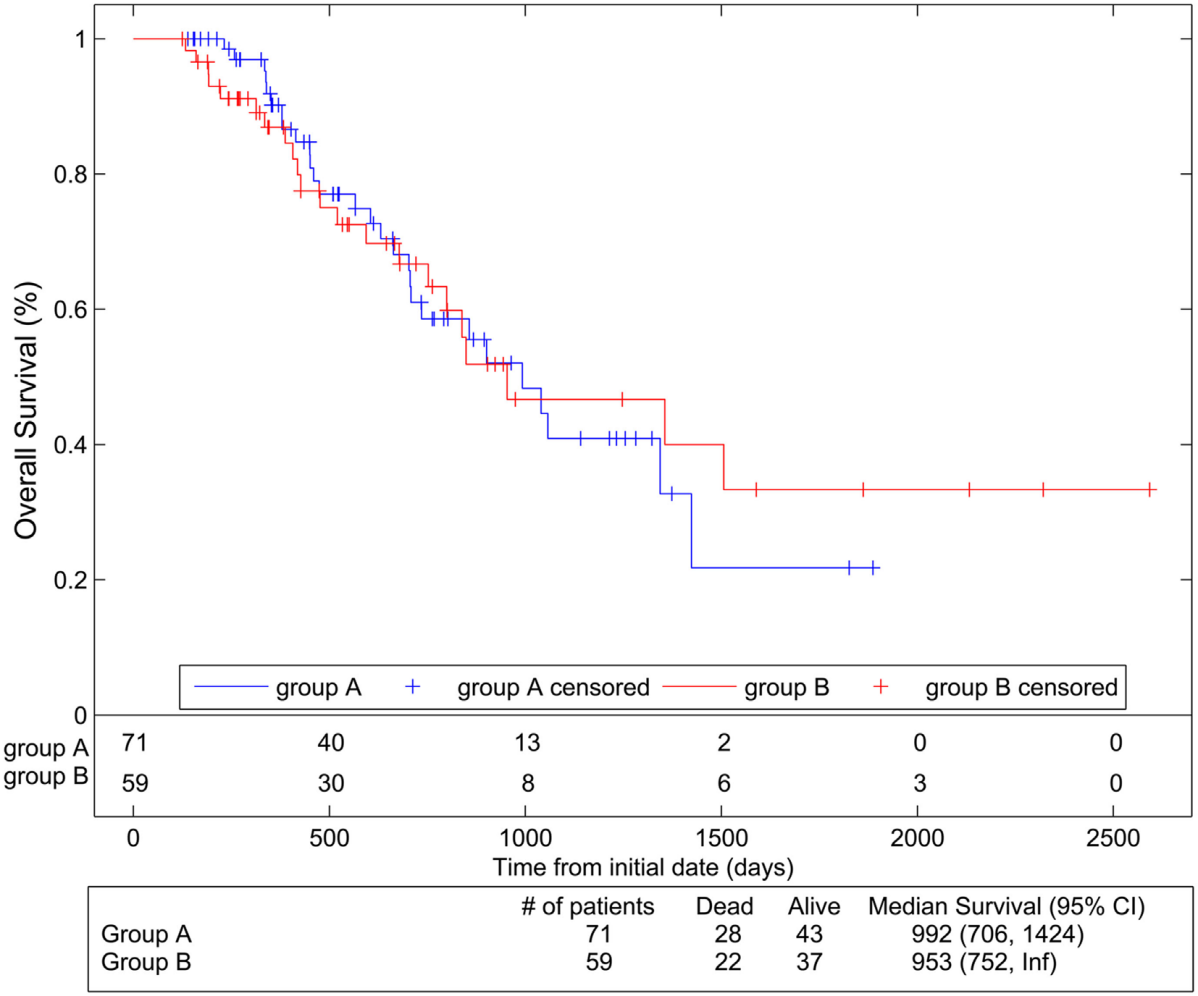
**Overall survival compared between the two arms using Kaplan–Meier analyses**. Group A represents Arm 1 (paclitaxel 15 mg/m^2^, three times per week), and Group B represents Arm 2 (paclitaxel 45 mg/m^2^ per week).

Data analyses were collected from patients receiving protocol treatments (71 in Arm 1 and 59 in Arm 2). Median follow-up was 23.4 months (range 7.4–85.2). Figure 2 shows Kaplan–Meier RFS comparison of the two arms. RFS was significantly better in Arm 1 than in Arm 2 [*p* = 0.005, HR 1.87 (95% CI 1.20, 2.90)]. The median RFS was estimated as 14.6 months (11.1–19.5) in Arm 1 vs. 9.4 months (8.4–13.3) in Arm 2. OS was not significantly different between arms [*p* = 0.91, HR 0.97 (95% CI 0.55, 1.70)] with a median 32.6 months (23.1–46.8) in Arm 1 and 31.3 months (24.7–Inf.) in Arm 2 (Figure 3).

### Tumor Response and Patterns of Failure

The total response rate for Arm 1 was significantly higher (83.1%) than that of Arm 2 (54.2%) (*p* = 0.001) (Table 2). The pattern of failure is as follows: local failure in the radiation port 11 (28.9%) in Arm 1 vs. 18 (42.9%) in Arm 2 (*p* = 0.08); chest failure, i.e., new lesions outside of radiation port 7 (18.4%) in Arm 1 vs. 8 (19.0%) in Arm 2 (*p* = 0.22); and distant failure 20 (52.6%) in Arm 1 vs. 16 (38.1%) in Arm 2 (*p* = 0.08). The differences in the patterns of failure between the two arms were not statistically significant.

**Table 2:** **Tumor response**.

Response rate	Arm 1 (*n* = 71)	%	Arm 2 (*n* = 59)	%	*p*-Value
	No. of patients		No. of patients		
Complete response	7	9.86	2	3.39	
Partial response	52	73.24	30	50.85	
Stable disease	8	11.27	23	38.98	
Progression	4	5.63	4	6.78	
Total response (CR + PR)	59	83.1	32	54.2	0.001

*Arm1, 15 mg/m^2^, three times a week; Arm 2, 45 mg/m^2^, once a week*.

### Toxicities and Safety

The observed toxicity data were collected for the two arms. Four treatment-related deaths were reported within 9 days of initiation of the study: one in Arm 1 and three in Arm 2. In Arm 1, the patient died of possible radiation pneumonitis with a complicated infection (*Pneumocystis carinii* infection, PCP). In Arm 2, two patients died of possible radiation pneumonitis with complicated infection and one died of heart failure and low sodium chloride.

Non-hematological toxicities during the CCRT phase for Arms 1 and 2 are listed in Table 3. None of the non-hematologic toxicities were statistically different between the two arms. Grade 3 or 4 hematologic toxicities are shown in Table 4. In Arm 1, 3 patients developed Grade 3 or 4 leukopenia vs. 15 patients in Arm 2 (*p* < 0.001). None of the patients developed Grade 3 or 4 neutropenia in Arm 1 in comparison to 10 patients in Arm 2 (*p* < 0.001).

**Table 3:** **Non-hematological toxicities**.

Toxicities	Arm 1 (*n* = 71) vs. Arm 2 (*n* = 59)	Grades (no. of patients)					*p*-Value
		G-1	G-2	G-3	G-4	G-5	
Weight loss	1	7	1	0	0	0	0.32
	2	10	1	0	0	0	
Fatigue	1	22	2	0	0	0	0.13
	2	14	7	0	0	0	
Fever	1	23	10	0	0	0	0.97
	2	18	8	0	0	0	
Nausea	1	8	10	0	0	0	0.34
	2	3	11	1	0	0	
Vomiting	1	1	1	0	0	0	0.55
	2	3	1	0	0	0	
Cough	1	28	9	0	0	0	0.47
	2	24	5	2	0	0	
Dyspnea	1	11	5	1	0	0	0.84
	2	6	4	1	0	0	
Esophagitis	1	15	21	3	0	0	0.11
	2	4	21	5	0	0	
Pneumonitis	1	1	8	13	0	1	0.52
	2	3	7	6	0	2	

**Table 4:** **Hematological toxicities: ≥Grade 3**.

Arms	Leucopenia		Neutropenia		Anemia		Thrombopenia	
Grades	G-3	G-4	G-3	G-4	G-3	G-4	G-3	G-4
1 (*n* = 71)	3	0	0	0	1	0	0	0
2 (*n* = 59)	14	1	8	2	2	0	2	1
*p*-Value	<0.001		<0.001		0.43		0.48	

## Discussion

Low-dose chemotherapy CCRT for LA-NSCLC has previously shown promising results with low toxicities by different investigators (15–17, 21–23). Among these, the best known is the randomized phase 3 study by Radiotherapy and Lung Cancer Cooperative Groups of the European Organization for Research and Treatment of Cancer, which compared very low-dose (6 mg/m^2^) daily cisplatin CCRT vs. weekly cisplatin (30 mg/m^2^) CCRT vs. RT alone for LA-NSCLC (21). Results demonstrated better survival of patients receiving daily low-dose cisplatin and RT than patients who received RT alone or weekly cisplatin. Such low doses of daily cisplatin in this context were thought to be the radiosensitizing effect of low-dose cisplatin in improving RT chest tumor control, which resulted in better survival while avoiding the toxicities of high-dose chemotherapy.

However, low-dose CCRT for LA-NSCLC has not been widely adapted, while poor chest tumor control of LA-NSCLC remains a major challenge for oncologists. Several single institution phase 1 and 2 studies have attempted RT dose-escalation to 74 Gy to improve chest tumor control and showed promising chest tumor control and survival (24–27). However, results of a phase 3 study (RTOG 0617) testing RT dose-escalation to 74 Gy vs. standard 60 Gy was disappointing (28). The median OS was 28.7 months (95% CI 24.1–36.9) for the 60 Gy arm and 20.3 months (17.7–25.0) for the high-dose 74 Gy arm (HR 1.38, 95% CI 1.09–1.76; *p* = 0.004). The exact reasons for the worse survival outcome in the 74 Gy arm remain to be elucidated. Nevertheless, the outcome of RTOG 0617 highlights the unresolved challenge in improving chest tumor control, and the need for novel strategies to enhance RT efficacy in the combined modality treatment of LA-NSCLC.

Pulsed low-dose paclitaxel was previously reported to be an effective radiosensitizing strategy (15–17). Chen et al. (17) reported that the kinetics of tumor shrinkage after pulsed low-dose paclitaxel CCRT were different from the standard conventional CCRT schedules, in that rapid tumor shrinkage occurred within 1 month after completion of CCRT, which is in contrast to the gradual tumor regression over a 5- to 6-month interval commonly observed (29). In addition to the rapid initial tumor responses, the infield (radiation port) tumor control was above 97% at 3 years. The exact mechanism of such rapid and effective tumor response and durable infield tumor control of pulsed low-dose paclitaxel CCRT is unknown, but one can theoretically attribute to the combined effects of paclitaxel G2/M cell cycle effect (9), its apoptotic effect (10, 11), tumor reoxygenation effect (30), and/or the antiangiogenic effect of low-dose chemotherapy delivered through metronomic dose schedules (31, 32). In the same context, we acknowledge that because the tumor response of standard treatment schedules is a gradual process, choosing any time point after CCRT to assess response rate is somewhat arbitrary and may be in favor of Arm 1. We chose the 1-month time point with the understanding of this issue. However, the RFS analyzed by the Kaplan–Meier method provided complementary information that was clinically more important than response rates, and Kaplan–Meier analysis would have accounted for the changes over a long period of time for both local tumor control and distant tumor control.

Our study reported significantly better tumor response rate, better RFS, and lower rates of Grade 3 and 4 leukopenia and neutropenia in the pulsed low-dose paclitaxel arm. The low rate of toxicities is consistent with the literature review on low-dose CCRT (5). We neither observed a significant difference in the patterns of failure of the two arms nor did we observe a significant difference in esophagitis or pneumonitis or other radiation-related acute side effects in the two arms. One may wonder why the better RFS in Arm 1 did not result in better OS, and if treatment-related toxicities may contribute to the lack of trend toward improving OS of Arm 1. We reviewed the Grade 5 toxicities of both arms, and there was only one Grade 5 lung toxicity in Arm 1 and two in Arm 2, thus Grade 5 toxicity did not contribute to the lack of OS benefit. Much larger sample sizes would have been necessary to reach any statistical power to detect the difference in OS.

We acknowledge that there are potential weaknesses of our study. The random number method is an assignment process in which all patients were assigned to either Group A or B completely at random, without monitoring the size of the groups. Our study design had planned to screen 134 patients with 67 randomized in each arm and to allow a 10% drop out rate. This design would have resulted in an estimated 60 patients in each arm for the final data analyses. Due to the method of random number table for the assignment of randomization, our study enrolled 74 patients in Arm 1 and 60 patients in Arm 2. In retrospect, we acknowledge that it would have been better to have a built-in process to balance assignments between the arms. However, despite the minor imbalance in randomization, the study resulted in sufficient number of cases in each arm for the study endpoint analyses (71 in Arm 1 and 59 in Arm 2), and there was no statistically significant imbalance of patient characteristics in the two arms (Table 2).

We chose to analyze our data based on patients completing the treatment regimen in the assigned arm instead of basing on the intention to treat (ITT). We think that final analyses of patients completing the treatment regimen in each arm have served the goal of our study better. The benefit of analysis based on ITT is to avoid effects from dropout and/or crossover, which may break the random assignment to the treatment groups in a study. Our study had very low dropout rates in both arms and did not allow for crossover, thus basing ITT for the analysis would not have made much difference.

Current practice standard is CCRT upfront for patients with good performance status, while induction chemotherapy followed by RT has been reserved for patients who cannot tolerate CCRT (33). A randomized phase 3 study did not demonstrate any benefit of adding induction chemotherapy to CCRT (34), and there is the lack of prior studies on induction chemotherapy followed by very low-dose CCRT for LA-NSCLC. We acknowledge that our study design is different from the current standard CCRT treatment regimens for LA-NSCLC due to logistical reasons and pattern of referrals in our region. Patients enrolled in our study had received 2–4 cycles of induction chemotherapy prior to enrollment. Because our primary goal was to assess low-dose chemotherapy in sensitizing chest RT to improve chest tumor responses, we designed the enrollment of patients to include the 2–4 cycles of cisplatin-based induction chemotherapy upfront, with the assumption that as long as all patients had received 2–4 cycles of cisplatin containing induction chemotherapy, the potential systemic effect on distant micrometastasis from induction chemotherapy would balance both arms. We acknowledge that it would have been helpful if the study were designed to include the induction chemotherapy followed by CCRT and had assessed tumor response to induction chemotherapy, but this was not feasible due to local practice and referral pattern.

In conclusion, our study showed that three times weekly paclitaxel (15 mg/m^2^) yielded better RFS and tumor responses, with lower hematologic toxicity than the weekly paclitaxel (45 mg/m^2^) schedule for CCRT in patients with LA-NSCLC who have received 2–4 cycles of induction chemotherapy. Based on this study, the survival of both arms was equivalent, but the low-dose arm experienced less toxicity. Whether low-dose paclitaxel CCRT translates to a survival benefit needs to be tested in a larger randomized study. In an era when maintaining quality of life during cancer therapy is important to patients, a regimen with low toxicity and better tumor control will be appropriate for those who cannot tolerate intense CCRT for LA-NSCLC.

## Ethics Statement

This study was carried out in accordance with the recommendations of the institutional research subject review boards of the participating hospitals, with written informed consent from all subjects. All subjects gave written informed consent in accordance with the Declaration of Helsinki.

## Author Contributions

HL: study design, patient enrollment, data collection and analysis, data interpretation, and drafting of manuscript; YC: study design, data examination, data analysis, major revision of the manuscript, and final critique of the manuscript; AS: patient enrollment, data collection, and data analysis; KP: provided input on chemotherapy, reviewed data, and review of manuscript; RY: patient enrollment and collection of data; YY: statistics; JL, HL, YW, TX, LF, HM, and JG: patient enrollment and collection of data; and GZ: study design, data analysis, and finalization of manuscript.

## Conflict of Interest Statement

The authors declare that the research was conducted in the absence of any commercial or financial relationships that could be construed as a potential conflict of interest.
